# Cardiovascular magnetic resonance characterization of left ventricular non-compaction provides independent prognostic information in patients with incident heart failure or suspected cardiomyopathy

**DOI:** 10.1186/s12968-014-0064-2

**Published:** 2014-10-02

**Authors:** Guha Ashrith, Dipti Gupta, Janel Hanmer, Robert M Weiss

**Affiliations:** Houston Methodist DeBakey Heart and Vascular Center, Suite Smith 1901, 6550 Fannin Street, Houston, TX 77030 USA; Division of Cardiology, Memorial Sloan-Kettering Cancer Center, New York, NY USA; Department of General Internal Medicine, University of Pittsburgh, Pittsburgh, PA USA; Division of Cardiovascular Medicine, Carver College of Medicine, University of Iowa, Iowa City, IA USA

**Keywords:** LV non-compaction, Cardiovascular magnetic resonance, Arrhythmias

## Abstract

**Background:**

With recent advances in imaging methods, detection of LVNC is increasingly common. Concomitantly, the prognostic importance of LVNC is less clear.

**Methods:**

We followed 42 patients (63% male, age 44 ± 15 years) with incident heart failure or suspected cardiomyopathy, in whom cardiovascular magnetic resonance (CMR) yielded a diagnosis of LVNC, for 27 ± 16 months.

**Results:**

LVNC was preferentially distributed among posterolateral segments, with apical predominance. Patients with maximum non-compacted-to-compacted thickness ratio (NC:C) < 3 improved by 0.9 ± 0.7 NYHA Class, compared to 0.3 ± 0.8 for patients with NC:C > 3 (p = 0.001). In 29 patients with baseline LVEF < 0.40, there was an inverse correlation between NC:C ratio, and the change in LVEF during follow-up. Tachyarrhythmias were observed in 42% of patients with LGE, and in 0% of patients without LGE (p = 0.02). In multivariate analysis, arrhythmia incidence was significantly higher in patients with LGE, even when adjusted for LVEF and RVEF.

**Conclusions:**

CMR assessments of myocardial morphology provide important prognostic information for patients with LVNC who present with incident heart failure or suspected cardiomyopathy.

**Electronic supplementary material:**

The online version of this article (doi:10.1186/s12968-014-0064-2) contains supplementary material, which is available to authorized users.

## Background

Isolated left ventricular (LV) non-compaction (NC) comprises a cardiac phenotype characterized by abundant muscular trabeculation overlying a thin layer of normal-appearing compacted LV myocardium. Early reports emphasized the rarity of the condition, familial inheritance, and high incidence of systolic dysfunction, malignant arrhythmias, and thromboembolic events, primarily in children [1].

More recently, LVNC has been recognized as a clinical phenotype with significant genetic [2] and prognostic heterogeneity [3]. Advances in imaging technology and increased diagnostic vigilance have led to more frequent detection of a non-compaction phenotype in adults, using criteria that vary between reports [4-6]. Predictably, the prognostic significance of a finding of LVNC varies widely, and may depend on the severity of co-existing structural heart disease [7-10]

The diagnosis of LVNC is usually made using a binary criterion, i.e. “present” or “absent”. However, the severity of hypertrabeculation, as well as the severity of thinning of underlying compacted myocardium, can vary widely among patients with LVNC. In addition, it is known that some patients demonstrate fibrosis in the compacted layer of myocardium [11,12], a finding that correlates with the severity of left ventricular systolic dysfunction [13]. However, the independent prognostic importance of myocardial fibrosis in patients with LVNC has not been reported.

We hypothesized that the severity and anatomic extent of LVNC, along with the presence of LGE, would correlate with clinical outcomes in patients with incident heart failure or suspected cardiomyopathy.

## Methods

This retrospective study was approved by the Institutional Review Board at the University of Iowa.

### Patient selection

We reviewed all adult cardiovascular magnetic resonance (CMR) clinical reports the University of Iowa Hospitals and Clinics between January 1, 2004 and March 30, 2011 (N = 994). Inclusion in the present study required a diagnosis of incident heart failure or a first presentation for suspected cardiomyopathy, and a CMR diagnosis of LVNC, based on the following criteria: i) non-compacted-to-compacted (NC:C) layer thickness ratio of ≥ 2.3 at end-diastole in at least two short-axis CMR slices, ii) absence of other congenital heart disease or coronary heart disease, iii) availability of follow-up clinical data. Flow limiting coronary artery stenoses were excluded by coronary angiography (N = 30), or by myocardial perfusion imaging (N = 12).

We identified 42 patients who met all study criteria. Four additional patients who met MRI criteria for LVNC, but for whom follow-up was not available, were excluded from further study. Patient records were reviewed for initial clinical presentation, family history, past medical history, NYHA functional class and medication use. Clinically indicated transthoracic echocardiography, which was not necessarily directed at detection of LVNC, was performed in all study subjects prior to CMR evaluation, and at least once during follow-up.

### Patient outcomes

Four categories of patient outcomes were assessed: change in LV systolic function, change in symptom class, incidence of tachyarrhythmias, and non-elective hospital admissions for cardiac causes.

Changes in LV systolic function were assessed by comparing echocardiographic measurements of LV ejection fraction (EF) at the time of CMR to subsequent echocardiographic LVEF at the time of latest follow-up. CMR measurements were not used for this comparison because a significant number of patients did not undergo follow-up CMR examination.

Changes in symptom status were ascertained from the electronic medical record, using New York Heart Association (NYHA) classification observed at the time of CMR study, and at the time of latest follow-up.

All hospital admissions for the study group were reviewed using the electronic medical record. Admissions for which the primary indication was a change in cardiac clinical status, e.g. heart failure or symptomatic arrhythmia, were included as endpoints. Admissions for non-cardiac indications, and elective admissions for cardiac causes, e.g. elective device implantation or anticoagulation bridging, were not included in this study endpoint.

Arrhythmias were ascertained from the electronic medical record, utilizing reports from electrocardiograms (ECG), ambulatory ECG monitors, and intracardiac device interrogation reports. For the present study, arrhythmias included supraventricular tachyarrhythmias, sustained or nonsustained ventricular tachycardia, and ventricular fibrillation. Sinus arrhythmias and electronically paced rhythms were not included in the arrhythmia endpoint.

### CMR acquisition

CMR was performed on a 1.5 Tesla scanner (Avanto^R^; Siemens, Erlangen, Germany) using a phased-array surface coil. Cine images were acquired using a True-FISP pulse sequence in long-axis planes and in contiguous 8-mm short-axis slices, which encompassed the whole heart. A total of 35/42 patients underwent imaging before, and 10 minutes after, administration of intravenous Gd-DTPA (0.1 mmol/kg), for assessment of late gadolinium enhancement (LGE). Images were acquired using inversion-recovery fast gradient-echo pulse sequences in short-axis, similar to the cine images. Inversion times were optimized individually to null normal myocardium.

### CMR image analysis

Ventricular volumes, mass, and systolic function were analyzed with QMass® MR 6.2.1 software (Medis, Leiden, Netherlands). LV volumes and mass were indexed to body surface area. Images were analyzed using the standard 16-segment model [14] in short-axis slices. The apical segment, which did not include any of the LV blood pool, was not included in the analysis. Quantitative LGE analysis was done by selecting a “normal” region of interest averaging 50 mm^2^ within a region unaffected by LVNC, usually in the interventricular septum. Regions which displayed signal intensity > 5 standard deviations above the mean signal intensity of the normal region were designated “positive” for the presence LGE, according to a previously published convention [13]. Trabeculations were excluded from the quantification of LGE due to difficulty of assessment of LGE close to the blood pool. Inter-observer variability was assessed for LVEDV, signal intensity of the region of interest used for assessment of LGE, and maximum NC:C ratio, by two observers blinded to one another’s findings.

Statistical analyses were performed using SPSS 20 software. The Chi-square test or Fisher’s exact test was used to analyze categorical variables (Fisher’s exact was used if the expected number of events in any cell was <5). Independent *t*-test was used to compare continuous variables between groups. Paired *t*-test was used to compare NYHA class before and after completion of the study period. Continuous variables are presented as mean ± SD, and categorical variables as N (%). Logistic multivariate regression with exact inference, two-way repeated measures ANOVA and Pearson Rank Correlation tests were used to assess the association between MRI characteristics and clinical outcomes. Intraclass correlation between raters was estimated using a two-way random effects model. All statistical tests were two-sided, with p-values ≤ 0.05 considered statistically significant.

## Results

Demographic, clinical, and CMR characteristics of the patients are listed in Table 1. Most patients were in NYHA Class II or III, and were receiving medications for heart failure, at study outset. Duration of follow up was 27 ± 16 months.

**Table 1:** **Baseline characteristics**

**Characteristics**	**n = 42**
Age*	44 ± 15
Caucasian, n (%)	37(88)
Male Gender, n (%)	27(64)
LVEF*	36 ± 15
RVEF*	44 ± 14
Maximum NC/C ratio*	3.6 ± 0.8
Number of NC segments*	5.9 ± 1.8
Initial NYHA Class, n (%)	
I	2(5)
II	14(33)
III	24(57)
IV	2(5)
% of LGE*	13 ± 12
LVEDVI(ml/m^2^)*	131 ± 36
LV Mass Index(gm/m^2^)*	62 ± 29
Medication Use, n (%)	
-β Blockers	33(79)
-ACE Inhibitors/ARBs	35(83)
-Loop Diuretics	21(50)
-Aldosterone Antagonists	16(38)
-Digoxin	10(24)

*Mean ± SE; ARBs angiotensin receptor blockers.

### CMR findings

**Figure 1:**
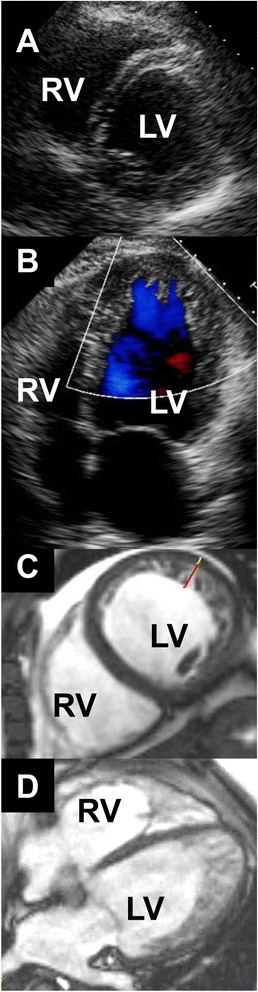
**Imaging left ventricular non-compaction in a single patient. A**: Short-axis echocardiogram at the level of the chordae tendineae, demonstrating normal-appearing myocardium. **B**: Apical 4-chamber echocardiogram acquired during early systole, depicting blood flow out of apical sinusoids (blue). **C**: Short-axis mid-ventricular CMR image, demonstrating abundant trabeculation overlying a very thin compacted myocardial layer in the lateral left ventricular (LV) wall. Red bar: trabecular thickness; white bar: compacted wall thickness. **D**: 4-chamber CMR image depicting LVNC extending from the apical septum clockwise to the lateral LV wall. RV: right ventricle.

Representative echocardiographic and CMR images from a patient with LVNC are shown in Figure 1.

#### Anatomical distribution of LVNC

**Figure 2:**
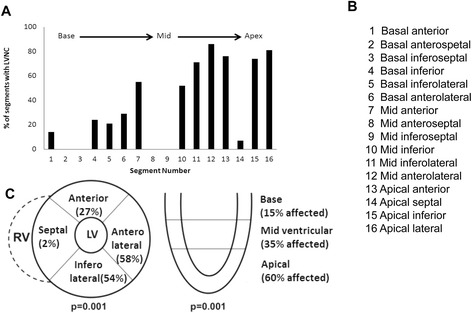
**Anatomical distribution of LVNC. A**: Frequency of LVNC among all 42 patients, according to the 16-segment model (ref. 14). **B**: Key to segment nomenclature (from ref. 14). **C**: Circumferential and longitudinal distribution of LVNC, grouped by region.

Non-compaction was present in 248/672 (41%) of myocardial segments, or 5.8 ± 1.8 segments per patient (range: 3-10). LV apical segments most often demonstrated non-compaction, and basal segments were least often affected. Circumferential distribution favored anterolateral and inferolateral segments, whereas septal involvement was rare (Figure 2).

#### Severity of LVNC

NC:C ratio was > 3.0 in 29/42 patients. At study entry, patients with NC:C > 3.0 also had greater anatomic extent of LVNC than patients with NC:C ≤ 3.0 (Table 2). Other clinical and imaging features were similar between patients with NC:C > 3.0 and those with NC:C ≤ 3.0.

**Table 2:** **Baseline characteristics in patients classified on the basis of severity of non-compaction**

**Characteristics**	**NC:C >3.0 (n = 29)**	**NC:C ≤ 3.0(n = 13)**
Age	44 ± 14	44 ± 18
Caucasian, n (%)	25(89)	12(92)
Male Gender, n (%)	21(72)	8(62)
LVEF	0.34 ± 0.17	0.37 ± 0.14
RVEF	0.45 ± 0.14	0.40 ± 0.14
% LGE in C-myocardium	11 ± 12	10 ± 11
Number of NC segments	6.3 ± 1.7	5.0 ± 1.8*
LVEDVI(ml/m^2^)	132 ± 36	127 ± 38
LV Mass Index(gm/m^2^)	70 ± 28	60 ± 29

*p < 0.05 vs. NC:C > 3.0.

#### Late gadolinium enhancement

**Figure 3:**
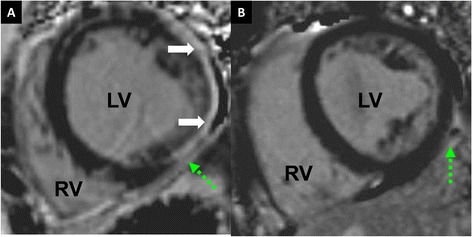
**Late gadolinium enhancement. A**: Short-axis phase-sensitive inversion recovery CMR image from a patient with extensive LGE (solid arrows). A still-frame True-FISP image can be viewed in Additional file 2: Figure S1. Contractile function in the region of myocardium with LGE can be viewed in the Additional file 1. **B**: Short-axis phase-sensitive inversion recovery image from a patient without LGE. Dashed arrows indicate epicardial fat. LV left ventricle; RV right ventricle.

**Figure 4:**
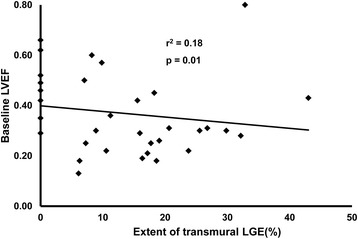
**Extent of transmural LGE vs. baseline global LV systolic function.** There is a modest inverse correlation between extent of LGE and CMR-derived LVEF.

LGE in compacted myocardium underlying LVNC was observed in at least one myocardial segment in 26/35 patients who received Gd-DTPA (Figure 3). There was a modest inverse correlation between the absolute amount of LGE and baseline LVEF (Figure 4; Additional file 1). Patients with LGE also had higher LVEDVI and LV mass than patients without LGE (Table 3).

**Table 3:** **Baseline characteristics in patients classified on the basis of presence of LGE**

**Characteristic**	**LGE present (n = 26)**	**LGE absent (n = 9)**
Age	42 ± 16	42 ± 12
Caucasian, n (%)	22(85)	9(100)
Male Gender, n (%)	21(81)	2(22)
LVEF	30 ± 12	48 ± 10*
RVEF	40 ± 13	51 ± 9*
Maximum NC/C ratio	3.6 ± 0.8	3.3 ± 0.8
Number of NC segments	6.0 ± 1.6	5.1 ± 2.0
LVEDVI(ml/m^2^)	141 ± 36	96 ± 21*
LV Mass Index(gm/m^2^)	72 ± 30	50 ± 23*

*p < 0.05 vs. LGE present.

#### Interobserver variability

Two observers blinded to one another’s findings performed measurements of the LVEDV, signal intensity of the region of interest used for assessment of LGE, and maximum NC:C ratio. There was very good agreement between observers for all three parameters (r^2^ > 0.90 for all; intra-class correlation = 0.99, 0.94 and 0.98 respectively (Additional file 2: Figure S2).

### Clinical outcomes

#### Changes in LV systolic function

Of the 29 patients with impaired baseline LV systolic function (echocardiography, LVEF < 0.40), 10 improved their LVEF by ≥ 0.10 during follow-up (echocardiography, 737 ± 476 days after initial CMR acquisition).

#### Change in cardiac symptom status

For all study patients, NYHA class was 2.6 ± 0.6 at study outset (range: 1 – 3), and 2.1 ± 0.7 at the time of latest follow-up (p < 0.001).

There were 34 non-elective hospitalizations for cardiac causes in 18 patients. Tachyarrhythmias were detected in 14 patients, 12 of whom had undergone imaging for LGE. Four patients had supraventricular arrhythmias, one had ventricular fibrillation, three had sustained ventricular tachycardia, and 6 had nonsustained ventricular tachycardia. Four patients underwent arrhythmia ablation. Twelve patients underwent ICD implantation for clinical indications, 288 ± 92 days after index MRI. Four patients experienced at least one appropriate ICD shock. There were no deaths, transplantation or ventricular assist device implantations during the follow up period.

### Relationship between CMR findings and clinical outcomes

#### Changes in LV systolic function

Baseline LVEF, LVEDV, and LVESV did not correlate significantly with the maximum severity of non-compaction (NC:C ratio; p ≥ 0.15 for each comparison) or the anatomic extent of LVNC (# affected segments; p = 0.30).

**Figure 5:**
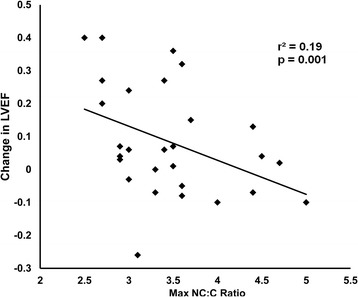
**Severity of non-compaction at baseline forecasts changes in echocardiographic LVEF during follow-up.** Data are shown for the 29 patients who had baseline LVEF < 0.40.

Receiver operating characteristics analysis identified an NC:C ratio of > 3.0 as candidate predictor of improvement in LVEF ≥ 0.10. There was an inverse correlation between maximum NC:C ratio, and change in LVEF during follow-up (r^2^ = 0.19, p = 0.001) (Figure 5). In patients with baseline LVEF < 0.40, there was a trend toward inverse correlation between LGE, and change in LVEF during follow-up (r = -0.35, p = 0.06).

#### Symptom status

**Figure 6:**
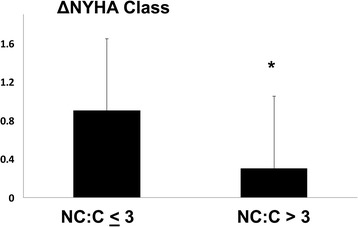
**Severity of non-compaction forecasts changes in symptom status.** Patients with less severe non-compaction were more likely to improve functional class than patients with more severe non-compaction. NC:C = maximal non-compacted layer thickness-to-compacted layer thickness ratio. NYHA Class = New York Heart Association Functional Class. *p < 0.05.

During follow-up, patients with maximum NC:C ratio ≤ 3 improved by 0.9 ± 0.8 NYHA Class, compared to 0.3 ± 0.9 for patients with NC:C > 3 (p = 0.001) (Figure 6).

#### Arrhythmia

**Figure 7:**
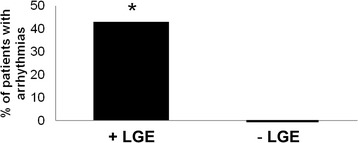
**Late gadolinium enhancement at baseline forecasts arrhythmias during follow-up.** +LGE refers to late gadolinium enhancement in compacted myocardium. *p < 0.05.

The anatomic extent of LVNC, grouped by quartiles, was not significantly correlated with the likelihood of arrhythmia. Patients with LGE in compacted myocardium were more likely to incur clinically significant arrhythmia, when compared to patients without LGE (42% vs. 0; p = 0.02; Figure 7). In separate logistic multivariate analyses, LGE remained a significant predictor of clinically significant arrhythmia even after adjusting for LVEF (LGE p = 0.03; LVEF p = 0.53), RVEF (LGE p = 0.01; RVEF p = 0.96), and both LVEF and RVEF (LGE p = 0.03; LVEF p = 0.39; RVEF p = 0.55). ICD shocks for ventricular tachycardia (n = 3) or ventricular fibrillation (n = 1) occurred in 4/9 patients with LGE who underwent ICD implantation, and in 0/3 patients without LGE who underwent ICD implantation (p = NS).

#### Hospital admission

Non-elective hospitalizations for cardiac causes were more likely in patients with LGE than in patients without LGE (0.8 ± 1.0 vs. 0.2 ± 0.3 per patient; p = 0.04).

## Discussion

The most important finding of this study is that, in patients with incident heart failure or suspected cardiomyopathy who receive a diagnosis of LVNC, CMR-based morphologic features of the LV impart important prognostic information with respect to recovery of LV systolic function, symptom status, and incidence of tachyarrhythmias. In patients with newly recognized or suspected cardiomyopathy, the ability to forecast responses to standard therapeutic or preventive measures would be clinically useful. For example, a decision to refer a patient for resynchronization or implantable cardioverter-defibrillator therapy can depend on responses to therapy over time. We found that changes in LVEF during follow-up were correlated, inversely, with the maximum severity of non-compaction. We also found that changes in symptom status correlated inversely with the severity of LVNC.

### Prognostic importance of LGE

Patients with the LVNC phenotype are putatively at risk of death, heart failure decompensation, arrhythmias, and transplantation. Two recent studies reported rates of heart failure hospitalizations (30-38%), arrhythmias (20-30%) in patients with the LVNC phenotype [4,7] which are comparable to our findings. However, the prognosis varies widely among patients with LVNC, which has tended to be reported as “present” or “absent” [15]. Our findings extend this body of knowledge, by stratifying the likelihood of hospitalization or arrhythmia, according to the presence or absence of LGE.

Our findings in patients with LVNC parallel previously reported trends for the broader population of patients with dilated cardiomyopathy, where the presence of LGE portends worse outcome [16,17]. Importantly, we found that the presence of LGE in patients with LVNC was an independent predictor of arrhythmia or non-elective hospital admission, whereas baseline LVEF was not, in our study group with restricted inclusion criteria.

#### Limitations

This study has the limitations of a retrospective study. There could be selection bias since the patients were seen in a tertiary care center and most of them were referred for incident heart failure. The sample size was small, but sufficient to provide some prognostic stratification. Links between CMR findings and clinical outcomes are presented as correlations or predictors. We are not able to prove causality of CMR findings for clinical outcomes, since treatment patterns may have varied between patients. Our findings are restricted to patients with incident heart failure or suspected cardiomyopathy, a cohort whose prognosis may be more diverse than patients with longstanding heart failure or those who with subclinical disease.

Diagnostic and therapeutic decisions were made on clinical grounds for all patients included in this retrospective study. Clinicians were aware of some CMR findings, which could potentially introduce an element of bias toward a conclusion that CMR is clinically useful. However, we did not observe more vigilant diagnostic follow-up in patients with more severe LVNC. For example, follow-up echocardiography was performed 542 ± 435 days after index CMR in patients with LGE vs. 585 ± 379 days in patients without LGE (p = NS). Ambulatory ECG-monitoring and ICD implantation were performed based on established clinical criteria, which did not invoke any specific findings from CMR. Most important, on clinical CMR reports, the NC:C ratio was not reported, and thus could not have influenced clinical decision-making.

By inclusion only of patients who met established criteria for LVNC, we are not able to determine the prognostic importance of LVNC per se, compared to patients without LVNC. However, we are able to identify group of patients, which have mild LVNC, limited anatomic extent of LVNC, and absence of LGE, who have a better prognosis with respect to clinical improvement, and who are at low short-term risk for arrhythmia or non-elective hospital admission.

## Conclusions

In patients for whom CMR provides a diagnosis of LVNC, there is considerable variability in the severity, anatomic extent, and association with LGE, each of which correlates with important clinical outcomes. Larger prospective studies are needed to determine whether these findings will be useful to guide therapeutic and preventive decisions in patients with LVNC.

## Additional files

Additional file 1:
**Short-axis cine True-FISP images from the patient depicted in Figure** 3 **A.** The region corresponding to dense LGE in the lateral LV wall demonstrates systolic thickening and endocardial shortening. Additional file 2: Figure S1. True-FISP image from the patient depicted in Figure 3A. Solid arrows delineate compacted myocardium. Dashed arrow indicates epicardial fat. LV left ventricle; RV right ventricle. Systolic function can be viewed in the Additional file 2. **Figure S2.** Interobserver variability. A: Signal intensity, used to determine presence or absence of LGE. B: Maximum NC:C layer thickness ratio. C: Left ventricular end-diastolic volume (LVEDV).

## Abbreviations

ICD: Implantable cardioverter defibrillator
LVNC: Left ventricular non-compaction
LGE: Late gadolinium enhancement
NC:C: Non-compacted to compacted layer thickness ratio
