# Supplementary material for: Cardiovascular magnetic resonance characterization of left ventricular non-compaction provides independent prognostic information in patients with incident heart failure or suspected cardiomyopathy
Source: J Cardiovasc Magn Reson. 2014 Oct 2;16(1):64. doi: 10.1186/s12968-014-0064-2 (PMC4181715; doi:10.1186/s12968-014-0064-2)
Supplement: Additional file 2: Figure S1. — True-FISP image from the patient depicted in Figure 3A. Solid arrows delineate compacted myocardium. Dashed arrow indicates epicardial fat. LV left ventricle; RV right ventricle. Systolic function can be viewed in the Additional file 2. Figure S2. Interobserver variability. A: Signal intensity, used to determine presence or absence of LGE. B: Maximum NC:C layer thickness ratio. C: Left ventricular end-diastolic volume (LVEDV). [file 12968_2014_64_MOESM2_ESM.ppt]

## Slide 1
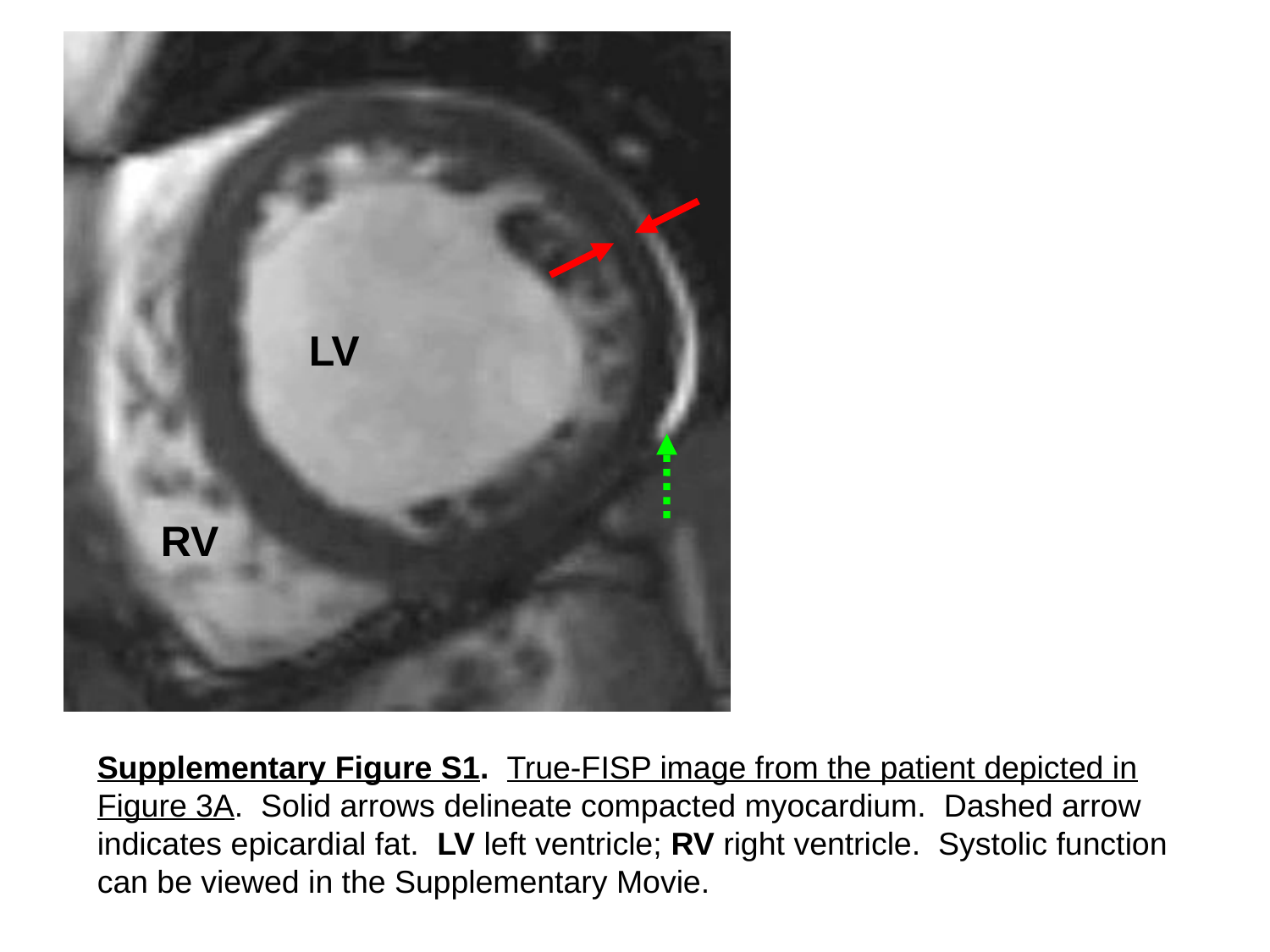

LV
RV
Supplementary Figure S1. True-FISP image from the patient depicted in Figure 3A. Solid arrows delineate compacted myocardium. Dashed arrow indicates epicardial fat. LV left ventricle; RV right ventricle. Systolic function can be viewed in the Supplementary Movie.

## Slide 2
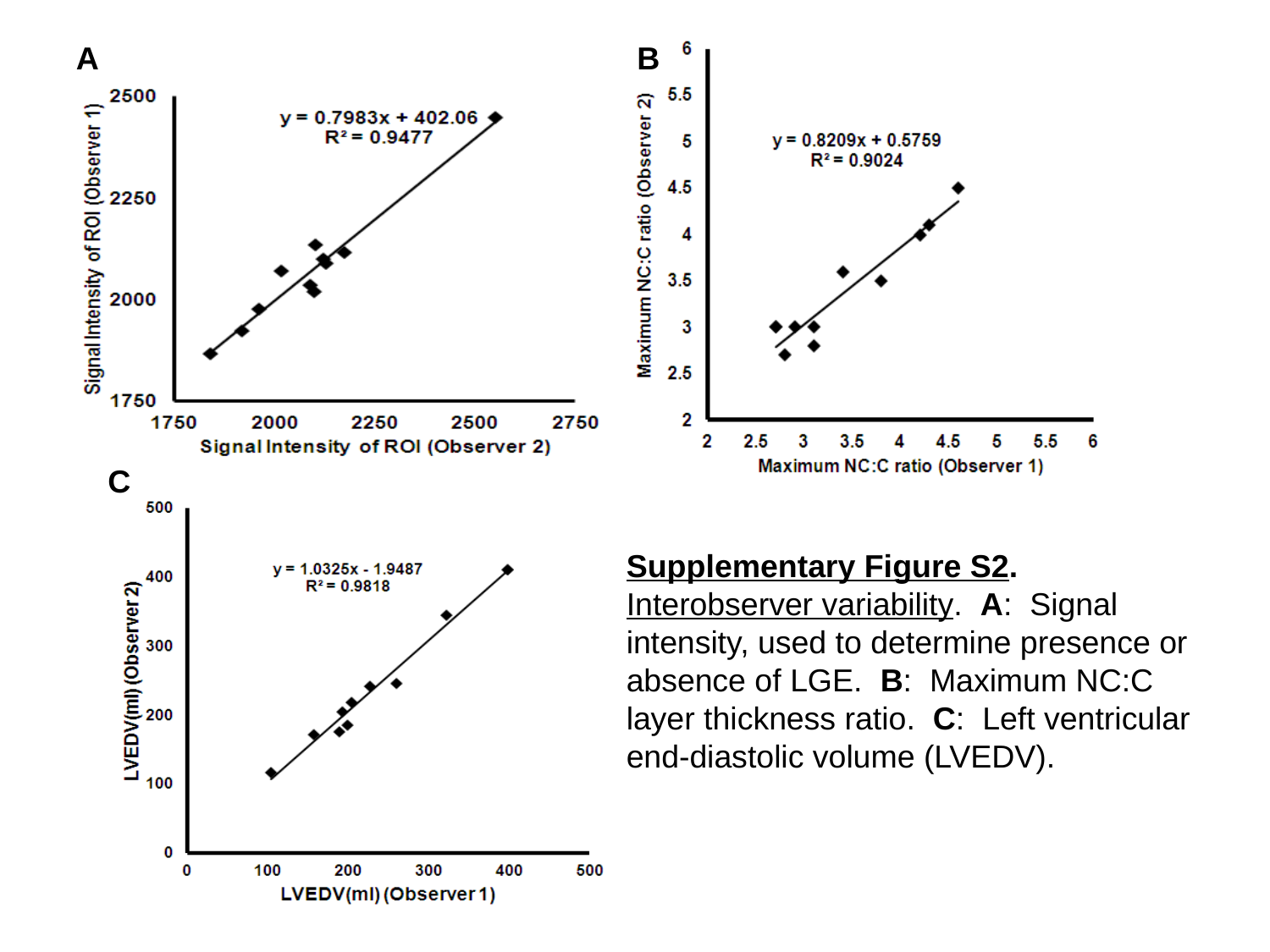

A
B
C
Supplementary Figure S2. Interobserver variability. A: Signal intensity, used to determine presence or absence of LGE. B: Maximum NC:C layer thickness ratio. C: Left ventricular end-diastolic volume (LVEDV).
